# Tumor Lysis Syndrome in a Retroperitoneal Sarcoma

**DOI:** 10.1177/2324709614542340

**Published:** 2014-07-04

**Authors:** Yousef Zakharia, Joshua Mansour, Srinivasa Vasireddi, Kais Zakharia, Eduard Fatakhov, Christopher Koch, Borys Hrinczenko

**Affiliations:** 1 Georgia Regents University, Augusta, GA, USA; 2 Michigan State University, East Lansing, MI, USA

**Keywords:** tumor lysis syndrome, retroperitoneal sarcoma, cancer

## Abstract

In the present case, a 49-year-old white female presented to the clinic with a 2-month history of nausea, vomiting, and right upper quadrant pain. On examination a 3-cm mass on the right anterior scalene muscle was noted. A computed tomography scan was performed revealing a 8.7 × 7.7 × 6.1 cm retroperitoneal mass with possible invasion of the inferior vena cava and right renal and left common iliac veins. An excisional biopsy was performed with pathology compatible with spindle cell sarcoma. The patient was then sent for follow-up at the sarcoma clinic as an outpatient. However, before chemotherapy was to be started the patient would be admitted to the hospital with progressively worse nausea and vomiting. At that time the patient’s lab work showed lactic acidosis, acute renal failure, hyperuricemia, hyperphosphatemia, and hypocalcemia, which met the Cairo–Bishop criteria for tumor lysis syndrome (TLS). The patient was admitted to the intensive care unit and kidney dialysis initiated. The patient would become progressively obtunded at which time the family opted for hospice care. The patient eventually succumbed peacefully 3 days after her last admission. In this case report, we briefly review the literature on TLS in solid tumors, and we present a rare case of spontaneous TLS in a retroperitoneal sarcoma.

## Background

Tumor lysis syndrome (TLS) is a medical emergency caused by massive lysis of malignant cells and release of intracellular components into the blood stream. This can cause numerous different electrolyte abnormalities, including hyperuricemia, hyperkalemia, and hyperphosphatemia, which result in deposition of calcium phosphate crystals in renal tubules. This deposition leads to the consumption of calcium and can cause obstructive nephropathy.^1^ Clinical and laboratory criteria for TLS have been proposed by the Cairo–Bishop definition in 2004, wherein laboratory TLS is defined by any change of 25% above or below normal for any 2 or more abnormal lab values (Table 1), within 3 days before or 7 days after the initiation of chemotherapy.^2^

**Table 1.** Initial Laboratory Data at Initial Diagnosis and at Readmission.

	Initial Diagnosis	At Readmission	On ICU Admission	% Change From Baseline
LDH (IU/L)	204	371		81.9
Alkaline phosphatase (IU/L)	88	239		171.6
Phosphorus (mmol/L)		6.9		
Potassium (mmol/L)	3.4	5.1		50.0
Blood urea nitrogen (mg/dL)	4	63	74.0	1750.0
Creatinine (mg/dL)	0.66	2.98	3.8	475.8
Calcium (mg/dL)	9	7.7		14.4
ALT (IU/L)	20	129		545.0
Uric acid (g/dL)	4.3	14.3		232.6
AST (IU/L)	20	164		720.0
Total bilirubin (mg/dL)	0.4	2.1		425.0
Albumin (g/dL)		3.1		
Lactic acid (mmol/L)		7.2	13.8	91.7

Abbreviations: ICU, intensive care unit; LDH, lactate dehydrogenase; ALT, alanine aminotransferase; AST, aspartate aminotransferase.

TLS occurs more frequently in hematologic malignancies such as Burkett’s lymphoma, especially after starting cytotoxic medications. However, spontaneous TLS has been reported in solid tumors like breast or small cell lung cancer.^3,4^ Indeed, in a literature review done by Baeksgaard and Sørensen,^5^ a total of 45 cases of TLS in solid tumors were found from 1977 to 2002. Another literature review done by Vodopivec et al^6^ reported a total of 100 cases of TLS in solid tumors between 1977 and 2011, with 2 cases of spontaneous sarcoma reported in Poland.^7^ In this report, we present a case of spontaneous TLS in retroperitoneal sarcoma.

## Case Report

**Figure 1.**
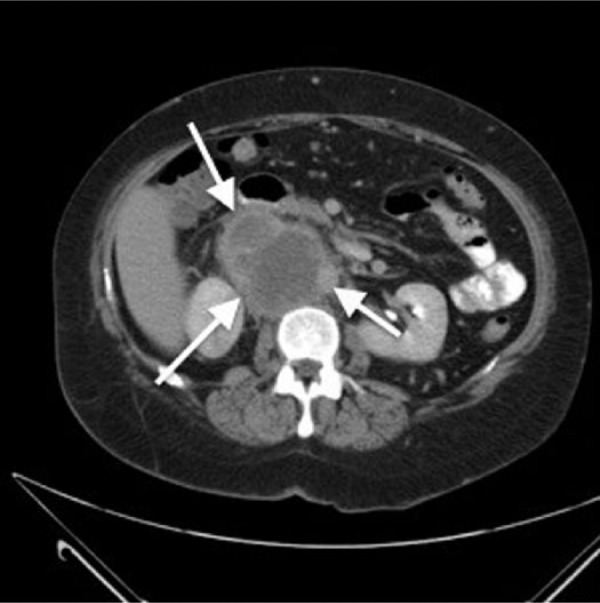
The retroperitoneal mass can be seen between the white arrows.

The patient was a 49-year-old white female who presented with a 2-month history of intermittent upper right quadrant pain (RUQ), nausea, and vomiting. The patient had a past medical history of hypertension but was otherwise healthy. On physical examination, the patient had mild RUQ abdominal tenderness with no palpable organomegaly. However, there was a 3-cm mass that was seen on the right anterior scalene muscle. After admission, initial lab work was within normal limits. An abdominal computed tomography scan showed a 8.7 × 7.7 × 6.1 cm retroperitoneal mass with possible invasion of the inferior vena cava and right renal and left common iliac veins, as seen in Figure 1. There was also a 1.3 cm lesion in the left lobe of the liver and 1.3 cm nodular density in the right base of the lung.

**Figure 2.**
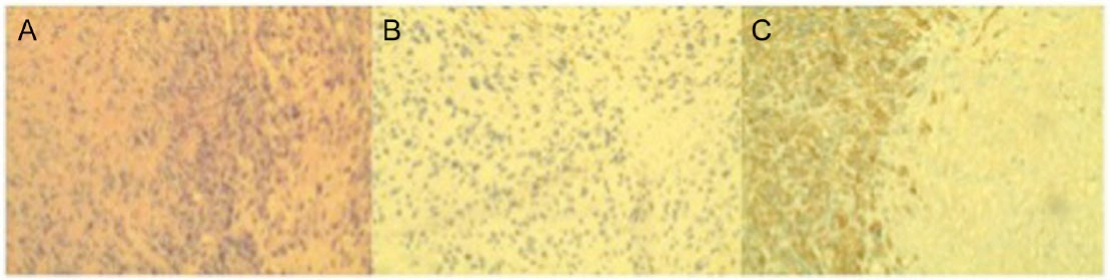
(A) The overall histologic appearance of a spindle cell tumor. (B) Positive staining for desmin. (C) Positive staining for caldesmon.

An excisional biopsy was performed that revealed an undifferentiated tumor suggestive of a spindle cell sarcoma. The tumor showed positive immunostaining for vimentin and caldesmon, with focal minimal positivity for desmin, as seen in Figure 2. While in the hospital the patient experienced a pulmonary embolus, which complicated her initial admission. At this time the patient was stabilized and discharged home to follow-up with the sarcoma clinic and start chemotherapy as an outpatient. However, the patient returned to the emergency room a few days before chemotherapy was to be initiated with worsening symptoms of nausea and vomiting.

Her lab work at this time revealed a uric acid of 14.3 g/dL, phosphorus of 6.9 mmol/L, potassium of 5.1 mmol/L, creatinine of 2.98 mg/dL, and a lactic acid of 7.2 mmol/L, at which time the patient was considered to be in spontaneous acute TLS (Table 2). The patient was taken to the intensive care unit and was started on aggressive hydration and rasburicase. The patient’s renal function subsequently continued to worsen, with her creatinine reaching a value of 3.8 mg/dL and her blood urea nitrogen reaching that of 74 mg/dL. The patient became obtunded, and even with hemodialysis her lactic acidosis worsened to 13.8 mmol/L. At this time, after a consultation with the health care proxy and family, the decision was made to initiate hospice care. The patient succumbed peacefully 72 hours after admission.

**Table 2.** Cairo-Bishop Criteria for Tumor Lysis Syndrome.

Element	Value	Change From Baseline
Uric acid	≥476 µmol/L (8 mg/dL)	25% increase
Potassium	≥6.0 mmol/L (or 6 mEq/L)	25% increase
Phosphorus	≥2.1 mmol/L (6.5 mg/dL) for children or ≥1.45 mmol/L (4.5 mg/dL) for adults	25% increase
Calcium	≤1.75 mmol/L (7 mg/dL)	25% decrease

## Treatment of TLS

Management of TLS includes aggressive hydration, treatment of the electrolyte imbalances, and correction of the acid–base disturbances. Commonly used pharmacological agents are allopurinol and rasburicase, which control the excess uric acid levels commonly present. Allopurinol, a competitive xanthine oxidase inhibitor, blocks the metabolism of hypoxanthine, xanthine to uric acid. Allopurinol decreases the new uric acid concentration, which further reduces the obstructive uropathy in patients who are at high risk for TLS. Allopurinol increases the serum xanthine and hypoxanthine levels in the serum, which could lead to acute renal failure by forming xanthine crystals in the renal tubules.^8,9^

An alternative option for treatment of TLS is lowering uric acid levels by facilitating conversion of uric acid to allantoin, which is more soluble in water. Rasburicase is recombinant urate oxidase, and the indications for use of rasburicase for adult population were expanded in 2009. Rasburicase contraindicated in pregnancy, lactating women, and glucose-6-phosphate deficiency. In comparison to allopurinol, it can be safely given in patients with renal insufficiency, and dosage adjustment is not needed. Uric acid concentration should be measured every 3 to 4 days after the final dose of treatment. If not followed closely, assay concentration may falsely appear low, which leads to early termination of rasburicase.^10,11^

## Conclusion

Although more common with hematologic malignancies, spontaneous TLS has been reported in solid tumors.^3,4^ Health care providers should be aware of this especially when there are risk factors. This includes but is not limited to a high tumor proliferation rate and high tumor burden (bulky tumor >10 cm, white blood cells >50 000/µL, pretreatment lactate dehydrogenase more than twice the upper limit of normal, organ and bone marrow involvement). When criteria for TLS are met by the Cairo–Bishop scale, aggressive efforts should be made to resolve these electrolyte imbalances promptly in order to avoid the multiple complications that may arise as a result. Of particular concern are the complications of renal failure that can be immediately life threatening. Moreover, when taken together TLS is a syndrome that can present in tumor of varying types, including solid tumors. Early recognition and aggressive treatment of TLS is pivotal for optimal patient outcomes.
